# Differential Expression of CD31 and Von Willebrand Factor on Endothelial Cells in Different Regions of the Human Brain: Potential Implications for Cerebral Malaria Pathogenesis

**DOI:** 10.3390/brainsci10010031

**Published:** 2020-01-06

**Authors:** Smart Ikechukwu Mbagwu, Luis Filgueira

**Affiliations:** 1 Anatomy Unit, Department of Oncology, Microbiology and Immunology, Faculty of Science and Medicine, University of Fribourg, 1700 Fribourg, Switzerland; 2 Department of Anatomy, Faculty of Basic Medical Sciences, Nnamdi Azikiwe University, 435101 Nnewi Campus, Nigeria

**Keywords:** cerebral microvascular endothelial cells, variations, blood-brain barrier, cerebral microcirculation

## Abstract

Cerebral microvascular endothelial cells (CMVECs) line the vascular system of the brain and are the chief cells in the formation and function of the blood brain barrier (BBB). These cells are heterogeneous along the cerebral vasculature and any dysfunctional state in these cells can result in a local loss of function of the BBB in any region of the brain. There is currently no report on the distribution and variation of the CMVECs in different brain regions in humans. This study investigated microcirculation in the adult human brain by the characterization of the expression pattern of brain endothelial cell markers in different brain regions. Five different brain regions consisting of the visual cortex, the hippocampus, the precentral gyrus, the postcentral gyrus, and the rhinal cortex obtained from three normal adult human brain specimens were studied and analyzed for the expression of the endothelial cell markers: cluster of differentiation 31 (CD31) and von-Willebrand-Factor (vWF) through immunohistochemistry. We observed differences in the expression pattern of CD31 and vWF between the gray matter and the white matter in the brain regions. Furthermore, there were also regional variations in the pattern of expression of the endothelial cell biomarkers. Thus, this suggests differences in the nature of vascularization in various regions of the human brain. These observations also suggest the existence of variation in structure and function of different brain regions, which could reflect in the pathophysiological outcomes in a diseased state.

## 1. Introduction

Cerebral microcirculation provides the anchor for the maintenance of brain functions through its supply of nutrients and gases in addition to the elimination of metabolic wastes from the brain [1,2]. The anatomo-physiological correlations of cerebral vascularization and the significant role of cerebral microvascular morphometrics determines cerebral hemodynamics and vascular responses [3]. These factors contribute to the regulation of cerebral microcirculation and are dependent on the local activities within different parts of the brain. In other words, regulation of cerebral circulation is influenced by autonomic, myogenic, local, and neuronal control [4]. The blood-brain barrier (BBB) is central to the regulation of cerebral microcirculation due to characteristic barrier properties and a transport system [1]. The BBB is principally composed of the cerebral microvascular endothelial cells (CMVECs), which form tight junctions together and are interlaced by astrocytes, pericytes, and a basal lamina [5]. These cells possess specialized receptor-mediated transport mechanisms and barrier properties [6,7], and equally contribute to the local control of cerebral microcirculation. To ensure the regulation of the local blood supply, CMVECs interact with other cells through paracrine signaling pathways to balance the distribution of oxygen and glucose. This suggests that the differences in the BBB in different regions of the brain could affect a localized functioning of the neural circuitry [7,8].

Impairment of the brain function results from a plethora of cerebrovascular events, including structural and functional dysfunction in cerebral hemodynamics. Cerebrovascular damage occurs in conditions such as stroke [9], cardiovascular [10] and neurodegenerative diseases [11], traumatic brain injury [12], and infectious diseases such as HIV [2] and cerebral malaria [5]. Cerebrovascular damage often involves obstruction of the BBB, which leads to its breakdown and impaired microcirculation. For example, in cerebral malaria, differential or preferential sequestration of parasitized red blood cells on the endothelial cells of the cerebral microvasculature have been suggested to trigger a series of events that initiate cerebrovascular damage. This has been reported as focal BBB breakdown with hemorrhages observed in autopsy studies on the cerebral tissues of cerebral malaria patients [13,14]. It is not clear if these differential or preferential sequestrations are due to the distribution of the endothelial cells within the different brain regions and or the distribution of the receptors involved in this process.

The distribution of endothelial cells in different human tissues such as the lungs, skin, bone marrow, lymph nodes, the heart, and the liver have been reported [15]. This study showed dynamic variations in the expression and distribution of the endothelial cell markers, which provides evidence of heteregeneity in the molecular features of endothelial cells and the phenotypical diversity, which it confers on the pattern of vascularization in different organs of the human body. Platelet endothelial cell adhesion molecule (PECAM-1) also known as cluster of differentiation 31 (CD31) and the von-Willebrand-Factor (vWF) are among the most common biomarkers used among others for the identification of endothelial cells in various tissues [15,16,17,18]. The paucity of literature on the characterization of brain endothelial cells in the different regions of the human brain and the evidence of cerebrovascular pathology in cerebral malaria patients prompted the need for this study.

Therefore, this study was undertaken with the aim to immunohistochemically investigate the expression of the most commonly known endothelial cell markers (CD31 and vWF) in different anatomical regions of the human brain. In our study, we identified differences in the expression of these biomarkers on the CMVECs. This could represent the phenotypic variation of CMVECs and could also reflect on the heterogeneity of the brain microcirculation and of the BBB. With a regional variation of vascular patterns, cellular and molecular characteristics between the white matter and gray matter regions of the brain, further investigations at both macro and molecular levels are needed to ascertain the regional differences in separate subregions of the brain. Understanding the nature and mechanism responsible for these differences would be relevant in generating appropriate in vitro models applicable in basic research with translational outcomes [19].

## 2. Materials and Methods

### 2.1. Acquisition of Human Tissue Samples

Human brain tissue blocks were obtained from three normal brains in a standardized protocol, always considering the same areas for the regions of interest. These regions were obtained from the following sites corresponding to the human brain map: median view of the precentral gyrus (area 4), postcentral gyrus (areas 3, 1, 2), visual cortex (areas 17 and 18), rhinal cortex (areas 28, 34, 35, and 36) and the hippocampus. The areas depicted on the precentral gyrus, postcentral gyrus, and visual cortex are illustrated in supplementary Figure S1. The tissues were fixed in 4% formalin in water and embedded into paraffin. The brains were obtained from the bodies of adults aged between 70–103 years. These bodies were received through the body donation program of Anatomy, University of Fribourg, Switzerland, following ethics regulations of Swiss Ethics (www.swissethics.ch) and the Swiss Academy of Medical Sciences, according to the Declaration of Helsinki. The body donors gave their informed consent. No information on the medical history of the donors were provided at the time of donation. The tissue samples and the corresponding histological images were anonymized.

### 2.2. Immunohistochemistry

#### 2.2.1. Single Staining

Paraffin-embedded sections obtained at 4 µm were deparaffinized in xylol and rehydrated serially in various grades of alcohol. The sections were hydrated and heated in a pressure cooker in citrate buffer 10 mM, pH 6, for 5 min. Sections were allowed to cool, and the slides were washed in phosphate-buffered saline. Endogenous enzymes were blocked with 1–3 drops of BLOXALL (Vector Laboratories, Burlingame, CA, USA) for 10 min, which is followed by blocking of unspecific bonding in 1–3 drops of 2.5% normal horse serum for 15 min. The slides were incubated with the primary monoclonal anti-mouse antibodies against human CD31 (ScyTEK Laboratories, West Logan, UT, USA) at 1:500 and rabbit anti-vWF (Diagnostic Biosystems, Pleasanton, CA, USA) at 1:200 overnight. Next, the samples (test and control) were washed and incubated for 30 min to 2 h with ImmPress reagents corresponding to the primary antibodies used. This was followed by a wash in buffer three times and subsequent incubation with freshly prepared AP-substrate ImmPACT Vector Red for 30 min. Nuclear counter staining with Hematoxylin QS was performed in 1–3 drops for 45 s. After washing with tap water, the slides were mounted on coverslips in VectaMount AQ (all reagents from Vector Laboratories) overnight. This staining method was considered appropriate for quantitative analysis of the expression of the biomarkers employed in this study.

#### 2.2.2. Double Staining of CD31 and vWF

This was performed in order to qualitatively analyze the co-expression of cd31 and vWF. For this, we used PolyStain DS Kit for Mouse and Rabbit antibody on Human tissue (DAB/Fast Red (Neobitech, Nanterre, France) Cat# NB-23-00089-1. The staining protocol used was performed following the manufacturer’s instructions.

### 2.3. Imaging and Analysis

The stained tissue sections were digitally captured at 20x magnification using NDP.scan, a high-speed slide scanner C9600 NanoZoomer-HT version 2.5. The digital images were analyzed using NIH ImageJwin64 software to determine the percentage area of expression of CD31 and vWF.

### 2.4. Percentage Area Quantification of CD31 and vWF Expression

Three different areas (fields of view at ×200 magnification) with clear expression and microvascular density in the grey and white matter of each of the brain regions used in the study were selected. From each of these images, five regions of interest (ROIs) were chosen at random (supplementary Figure S2). In the ROIs, the measurement of percentage area of these biomarkers’ expression was performed using “Measure percentage area” function on Image J. Before the measurement, the images were processed by color deconvolution using a set of macros instruction. The deconvoluted images were smoothened and thresholded. The threshold was set independently based on the threshold results for each image. The percentage area obtained was defined as a percentage of the area of CD31 or vWF-positive microvessels to the area of the tissue (CD31 or vWF area/area of tissue). This study did not take into consideration the number and size of vessels present in the tissue sections.

### 2.5. Statistical Analysis

All analyses were conducted with GraphPad Prism version 8.3.0. Variables were expressed as means ± SEM. One-way ANOVA with Tukey’s post comparison tests and two-tailed Student *t* tests were used to analyze differences between populations where appropriate. A *p* value < 0.05 was considered significant.

## 3. Results

**Figure 1:**
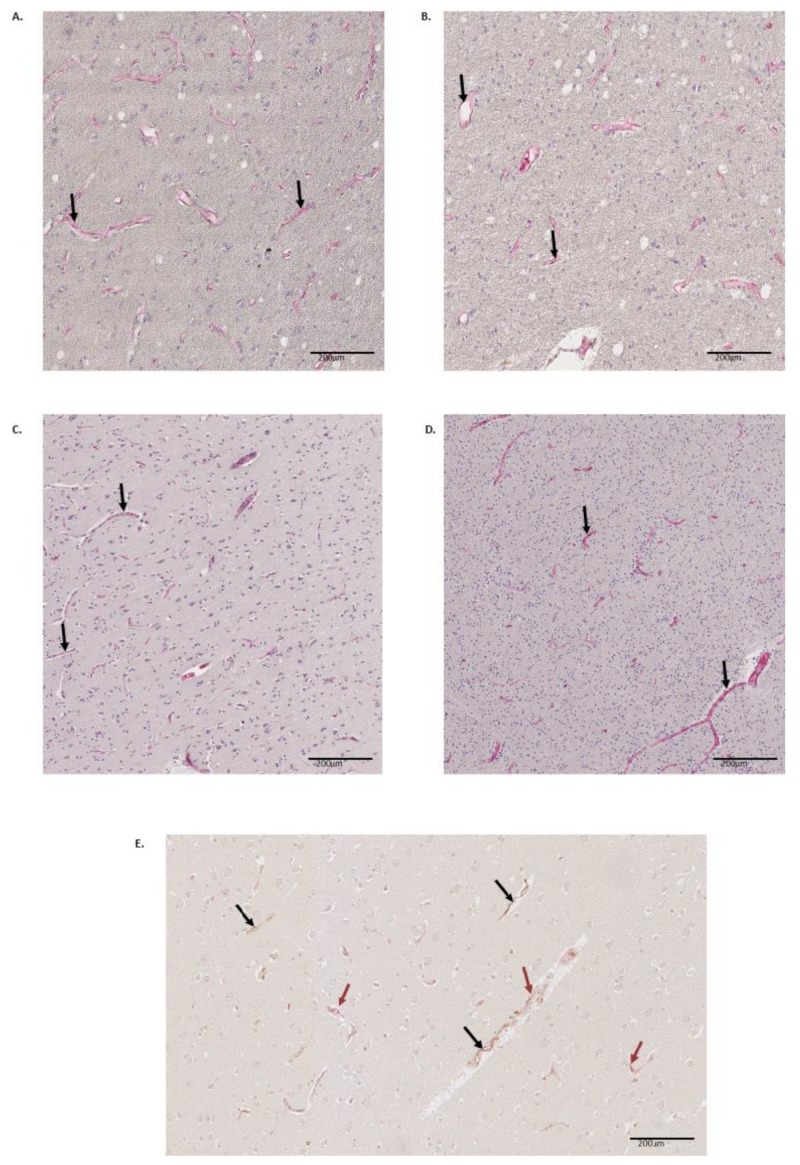
Expression of CD31 and vWF by cerebral microvascular endothelial cells. The cerebral microvascular endothelial cells expressed the biomarkers (black arrows) in different regions of the brain. The images are representative illustrations of the immunoreactivity of CD31 (**A**, **B** grey and white matter of precentral gyrus ×400) and vWF (**C**, **D** grey and white matter of the rhinal cortex ×400). Co-expression of CD31 and vWF in the hippocampus (**E** grey matter of hippocampus, ×800, black arrow indicates CD31 expression and red arrow indicates vWF expression).

Five different regions from three brains were investigated using an immunohistochemical demonstration to visualize differences in microvascular properties in response to CD31 and vWF staining. Our findings revealed that the test samples exhibited microvessels with distinctive delineated endothelial cell linings that expressed brain endothelial cell biomarkers CD31 and vWF in the different brain regions. The expression was higher in the grey matter than in the white matter of the regions investigated and the pattern of expression varied in the different brain regions as illustrated in the representative images (Figure 1A–D). Co-expression of the two markers were observed in the hippocampus, which indicated that they may be co-expressed by microvascular endothelial cells. However, various patterns of co-expression were found (Figure 1E). Quantitation of the expression of the biomarkers.

Expression of the two biomarkers was quantified on single stained sections for the three brains and the corresponding five brain regions. The two markers varied in their expression as quantified using a percentage area of expression. The expression of CD31 was highest in the precentral gyrus and vWF expression was highest in the visual cortex. Expression of both markers was higher in the grey matter than in the white matter of the regions investigated with statistically significant differences with the exception of the precentral gyrus in the expression of CD31 (Table 1).

**Figure 2:**
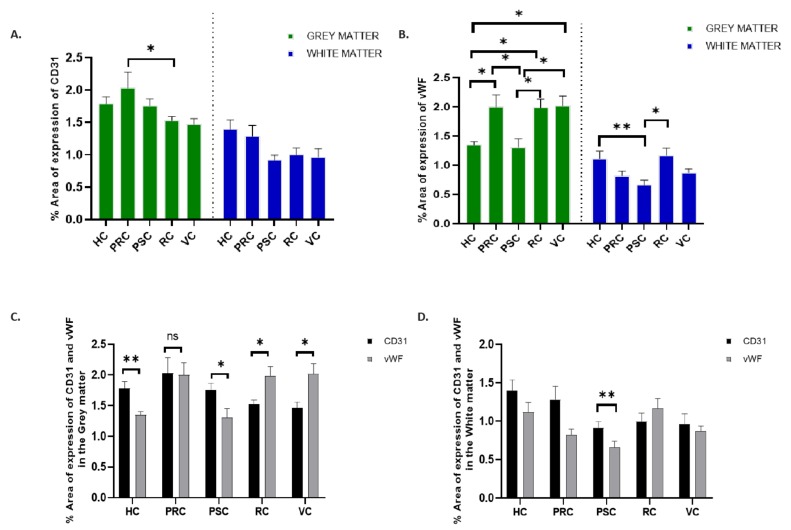
Quantification of the percentage area of expression of CD 31 and vWF. There was a significant difference in the expression of CD31 only in the grey matter between precentral gyrus and rhinal cortex (**A**). Significant differences were observed more in vWF expression in the grey matter of all the regions (**B**). Stronger differences in the expression of vWF in the white matter occurred between the hippocampus and the postcentral gyrus. On comparing the expression of both biomarkers, there was equal expression of both biomarkers in the grey matter of the precentral gyrus (**C**) while the expression of vWF was dominant in the visual and the rhinal cortex. In the white matter (**D**), vWF expression was higher in the rhinal cortex only and there was a statistically significant difference between both biomarkers in the postcentral gyrus. HC = hippocampus. PRC = Precentral cortex. PSC = Post central cortex. RC = rhinal cortex. VC = visual cortex. Error bars represent mean ± SEM. * *p* ≤ 0.05. ** *p* ≤ 0.001. (n = 15; 3 patients, 5 regions of interest).

For CD31 (Figure 2A), a higher percentage area of expression was found in the precentral region, which was followed by the hippocampus and postcentral gyrus. A statistically significant difference was only found to exist in the grey matter between the postcentral gyrus and the visual cortical region (*p* < 0.0389). In the white matter, there was no statistically significant difference between the regions.

For vWF (Figure 2B), a higher percentage area of expression was found in the visual cortex, precentral gyrus, and the rhinal cortex. Statistically significant differences were observed in the grey matter comparing the following regions: hippocampus versus precentral gyrus (*p* = 0.0240), hippocampus versus rhinal cortex (*p* = 0.0279), hippocampus versus visual cortex (*p* = 0.0196), postcentral gyrus versus precentral gyrus (*p* = 0.0145), postcentral gyrus versus rhinal cortex (*p* = 0.0170), and postcentral gyrus versus visual cortex (*p* = 0.0118). In the white matter, the statistically significant difference was observed between the hippocampus and the postcentral gyrus (*p* = 0.0118) and also between the postcentral gyrus and the rhinal cortex (*p* = 0.0032).

In comparing both biomarkers, the expression of CD31 and vWF was highest in the grey matter of the precentral gyrus (Figure 2C) with little or no significant difference in the percentage area of expression. CD31 was higher in the hippocampus and postcentral gyrus while vWF was higher in the visual and rhinal cortices.

The differences in these areas were statistically significant (Hippocampus (*p* = 0.0092), postcentral gyrus (*p* = 0.0192), rhinal cortex (*p* = 0.0371), and visual cortex (*p* = 0.0287)). In the white matter (Figure 2D), CD31 was higher in almost all the regions except for the rhinal cortex. The differences in the expression of both biomarkers was statistically significant in the white matter of the postcentral gyrus only (*p* = 0.0085).

## 4. Discussion

The Blood brain barrier lacks uniformity throughout the brain as a result of the variation in the structure and function of its components especially the CMVECs [5]. The specialized features of the CMVECs confers on them the ability to maintain the integrity of the BBB. Any alteration in the normal milieu of the CMVECs such as shear stress and increased production of reactive oxygen species would result in the dysfunction of the CMVECs and a further impact on the BBB [20].

CD31 and vWF are well-known biomarkers for detecting endothelial cells including CMVECs. They have been characterized in other organs of the human body such as the lungs [15]. We observed significant differences in immunoreactivity of CD31 and vWF between the grey matter and white matter of the different brain regions investigated in our study. This immunoreactivity varied across the brain regions. The variation was significant between some regions in either the gray matter or the white matter. Significant differences in expression patterns were also observed when both biomarkers were compared together in both the grey matter and the white matter. The characterization of CD31 and vWF expression on CMVECs in the normal human brain has rarely been addressed. These differences in the expression pattern of these biomarkers as seen in our study suggests that the BBB could display varying functions at different areas along the microvascular tree or regions of the brain [21]. This could also be related to the observation of heterogeneity in the expression profiles of BBB-related genes corresponding to CD31 and vWF [22].

The strong and clear positive expression of these biomarkers validates CD31 and vWF as biomarkers for the detection of CMVECs, which have been described in different studies [23,24,25]. Although we did not report the microvessel density in this study, the histological observation of the expression profile of these biomarkers suggests enriched microvasculature in the grey matter compared to the white matter, which have been reported in other studies. This characteristic difference is attributable to the synaptic and metabolic activities within these regions [21].

In addition to factors such as the agonal state, tissue sampling, and unknown existing pathology, the age of the brain samples used in our study could be considered a limitation to our study in generalizing our findings. This notwithstanding, age-associated microvascular changes have been implicated in Alzheimer’s disease (AD) and vascular dementia [26]. These changes have been suggested to influence the nature and function of the BBB [27,28]. They include loss of junctional complexes, inflammation, cellular damage, and vascular remodeling [29]. The age-related cellular damage results in cellular senescence, which affects functionality of the BBB [30]. On the other hand, hypertension-induced microvascular changes have been reported to cause alterations in the cerebral microcirculation, which results from neurovascular uncoupling, capillary rarefaction, and the disruption of the BBB [30]. The precise mechanisms for these observations have not yet been clarified. They are also believed to contribute to the development of microhemorrhages within the brain as well as in the pathogenesis of vascular cognitive impairment. As observed in our study, the decrease in the expression of the biomarkers in the white matter compared to the grey matter could also corroborate the susceptibility of the white matter to age-associated hypoperfusion in AD, which induces white matter lesions and reduction in cerebral blood flow [31]. The maintenance of cerebral microcirculation is influenced by neuroangiogenesis [32]. This gradually decreases with aging and have often been responsible for vascular cognitive impairment seen in Alzheimer’s disease. Cerebral capillary density is usually considered for this assessment. Sparse data on normal human-aged brains exist.

Age is considered a risk factor in the outcome of certain infectious conditions. For example, the outcomes of severe malaria infection revealed the highest number of cerebral complications among individuals 70 years old and older [33]. Therefore, in this context, we suggest, from our findings, that the differential expression of CD31 and vWF may contribute to specific sensitivity of some brain regions resulting in microvascular pathological changes. Differential expression of the endothelial biomarker between the grey matter and white matter can be attributed to the heterogeneity of biochemical and structural composition of the blood brain barrier including the CMVECs [8,34]. In silico and in vitro studies associated the difference in the expression of BBB-related molecules between the white matter and grey matter with the heterogeneity in CMVECs [5]. These differences could be associated with the differences between the expression of the investigated markers in the grey matter and the white matter in pathological conditions involving the activation of brain endothelial cells and/or dysfunction of the brain microvasculature [19]. In cerebral malaria, hemorrhages associated with an increase in fibrin accumulation were reported to be higher in the white matter [35,36,37,38]. In multiple sclerosis, white matter lesions involve disruption of the BBB and infiltration of immune cells into the brain parenchyma, which does not occur in grey matter lesions [39,40].

CD31 exhibits mechanosensory properties, which regulates vascular integrity and migration of immune cells [41] in response to changes in osmolarity and varying blood flow [42,43,44,45]. The pattern of expression of CD31 observed in our study implies a variation in the integrity of the BBB in the various regions of the brain. This is buttressed by the fact that CD31 is predominantly expressed at interendothelial junctions [46]. The expression of CD31 could increase in response to stimuli such as inflammation [47]. For example, the BBB dysfunction was associated with an increase in the expression of CD31 in the brain endothelium of cerebral malaria patients. The study did not report any difference between the grey matter and the white matter [48]. Furthermore, interferon gamma (IFN-γ), which is highly produced during malaria infection, induces the redistribution of CD31 from interendothelial junctions to the endothelial cell surface. This promotes the sequestration of infected red blood cells to the microvascular endothelium [49]. Sequestration of infected red blood cells in the brain endothelium has been considered the hallmark of cerebral malaria, which triggers a series of events leading to endothelial cell activation. Consequently, this culminates in the disruption of vascular integrity and results in increased vascular permeability, infiltration of brain parenchyma with leukocytes, and increased vascular leakage [50]. Although there are challenges in reporting precise molecular characterization in the nature of BBB damage in human cerebral malaria [51], this assertion that CD31 could play a role in cerebral malaria has been demonstrated by the association of CD31 polymorphisms with susceptibility to cerebral malaria [52]. Thus, we can speculate that there is regional variance in the maintenance of the BBB mediated by CD31. Thus, the modulation of BBB integrity in response to injury such as inflammation or stress [53], especially in the grey matter, still needs to be further characterized. 

vWF expression is well expressed on CMVECs [54]. Its varied expression offers an implication for modelling the functions of the BBB [22]. Permeability of the BBB is regulated by vWF as reported by Suidan and colleagues [54]. Endothelial cell-derived vWF has also been reported to play a role in thrombosis and proinflammation in stroke and in ischemic injuries [55]. Similar to CD31, vWF also modulates the function of the BBB in response to inflammatory stimuli [56]. The pattern of variation observed in the expression of vWF in our study suggests possible areas to consider when investigating the regulation vWF in various neuropathological conditions. For example, insults or injury on the microvascular endothelium causes the release of vWF thereby promoting vascular damage [54,57]. Additionally, vWF is upregulated in malaria infection and have been associated with malaria severity caused by *Plasmodium falciparum* [58]. The release and circulation of vWF implies activation of endothelial cells mediated by the interaction between endothelial receptors and parasite-derived molecules. Weibel-Palade (WP) bodies are intracellular storage organelles resident in endothelial cells. They store vWF, which is released upon endothelial cell activation. When released, vWF induces platelet recruitment to the surface of the endothelial cells to foster the cytoadherence of malaria-infected red blood cells to the endothelium. This cytoadherence promotes activation of the coagulation cascade. The overall outcome of this action is induction of proinflammatory responses that alter structure and function of the endothelial cells [59]. Intravascular aggregation of platelets have been reported to usually occur following shear stress on the arteriolar component microcirculation [60]. 

The observation from our double staining, seems to account for the differential phenotypic speciation in the expression of these biomarkers. This also suggests that the differential expression of CD31 and vWF could be responsible for making certain brain regions more susceptible to tissue damage in certain conditions, e.g. stroke [61,62] or cerebral malaria [52,63].

Furthermore, the association of vWF as markers of cerebral small-vessel disease, white matter hyperintensities and microhemorrhages [24,64] also poses another limitation on our study in addressing if our results could be different from pathological studies as there was no information on the medical history of the body donors used in our study. It is a challenge to characterize the difference between age and disease-related changes in BBB in healthy individuals as this heavily depends on the post-mortem brain tissues [65]. However, brain imaging using magnetic resonance imaging reported BBB breakdown during normal aging in the hippocampus [29]. 

Despite the limitations, our study provides some information that could be considered as a baseline for comparative investigations involving neurodegenerative diseases. This is also relevant for future studies to further characterize the pathogenesis of cerebral malaria in an elderly populations following evidence of age-related susceptibly to severe complications of malaria [66,67].

## 5. Conclusions

This study is the first to provide evidence of regional variation in the expression of brain endothelial cell biomarkers in the human brain by immunohistochemistry. The variation in the expression of CD31 and vWF suggests that there are differences in the properties of cerebral microvascular endothelial cells in different areas of the human brain. This differential expression pattern might influence the integrity and permeability of the BBB in different brain regions. Therefore, we suggest investigation of additional brain endothelial cell biomarkers, characterization of phenotypic characteristics of brain endothelial cells, and variation in the expression of tight junctional proteins and other factors, which are implicated in the function of the BBB, including ephrin B2, Ephrin type-B receptor 4 (EphB4), and vascular endothelial growth factor-A (VEGF-A).

## Figures and Tables

**Table 1:** Percentage area of expression of CD31 vWF by endothelial cells in five different brain regions. The two markers varied in their expression as quantified. The expression of CD31 was highest in the precentral cortex and vWF expression was highest in the visual cortex. Expression of both markers was higher in the grey matter than in the white matter with statistically significant differences, except for the precentral central cortex for the expression of CD31. (n = 15, three patients, five regions of interest).

Biomarkers	CD31 (Mean ± SEM)			vWF (Mean ± SEM)		
Brain Regions	Grey Matter (%)	White Matter (%)	*p* Values	Grey Matter (%)	White Matter (%)	*p* Values
*Hippocampus*	1.782 ± 0.11	1.394 ± 0.14	0.0105	1.346 ± 0.06	1.116 ± 0.13	<0.0001
*Precentral cortex*	2.032 ± 0.23	1.286 ± 0.17	0.1408	1.998 ± 0.20	0.8186 ± 0.08	<0.0001
*Postcentral cortex*	1.759 ± 0.11	0.918 ± 0.08	<0.0001	1.310 ± 0.14	0.6622 ± 0.08	0.0011
*Rhinal cortex*	1.527 ± 0.07	0.996 ± 0.11	<0.0001	1.987 ± 0.15	1.169 ± 0.13	<0.0001
*Visual cortex*	1.466 ± 0.90	0.964 ± 0.13	<0.0001	2.013 ± 0.17	0.8761 ± 0.06	<0.0001

## Supplementary Materials

The following are available online at https://www.mdpi.com/2076-3425/10/1/31/s1. Figure S1: The areas of the brain that were selected for the study, Figure S2: Illustration of Quantification of percentage area of expression of the biomarkers.
