# Supplementary material for: Differential Expression of CD31 and Von Willebrand Factor on Endothelial Cells in Different Regions of the Human Brain: Potential Implications for Cerebral Malaria Pathogenesis
Source: Brain Sci. 2020 Jan 6;10(1):31. doi: 10.3390/brainsci10010031 (PMC7016814; doi:10.3390/brainsci10010031)
Supplement: Supplementary file 1 [file brainsci-10-00031-s001.pdf]

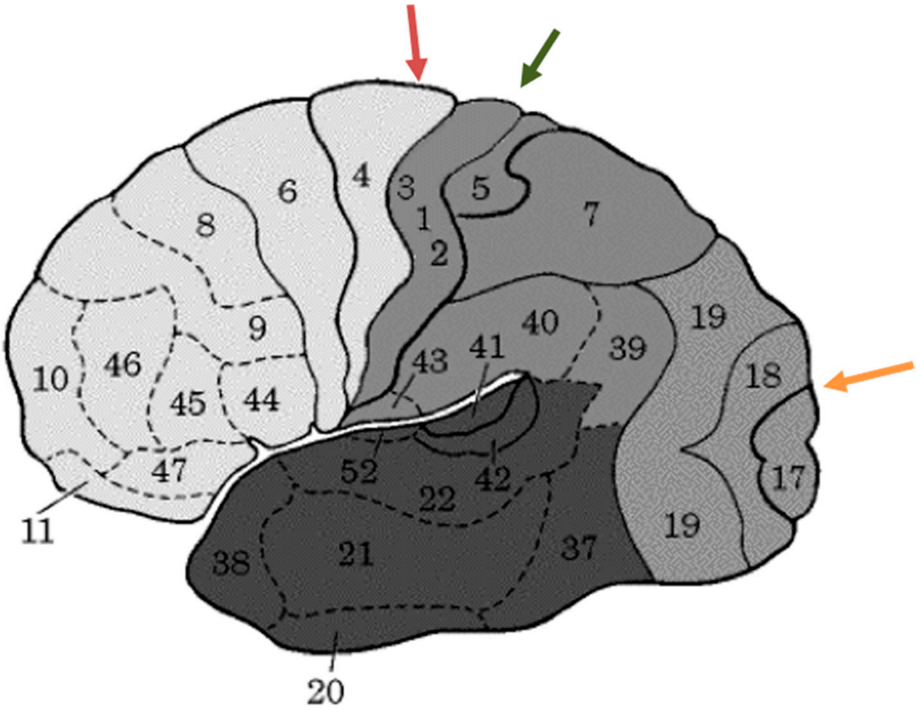

Figure S1: The areas of the brain that were selected for the study. These regions were obtained from the following sites corresponding to the human brain map as follows: median view of the precentral gyrus(area 4: red arrow),postcentral gyrus (areas 3,1,2;green arrow), Visual cortex (areas 17 and 18). The rhinal and the hippocampal regions are not shown in this image.

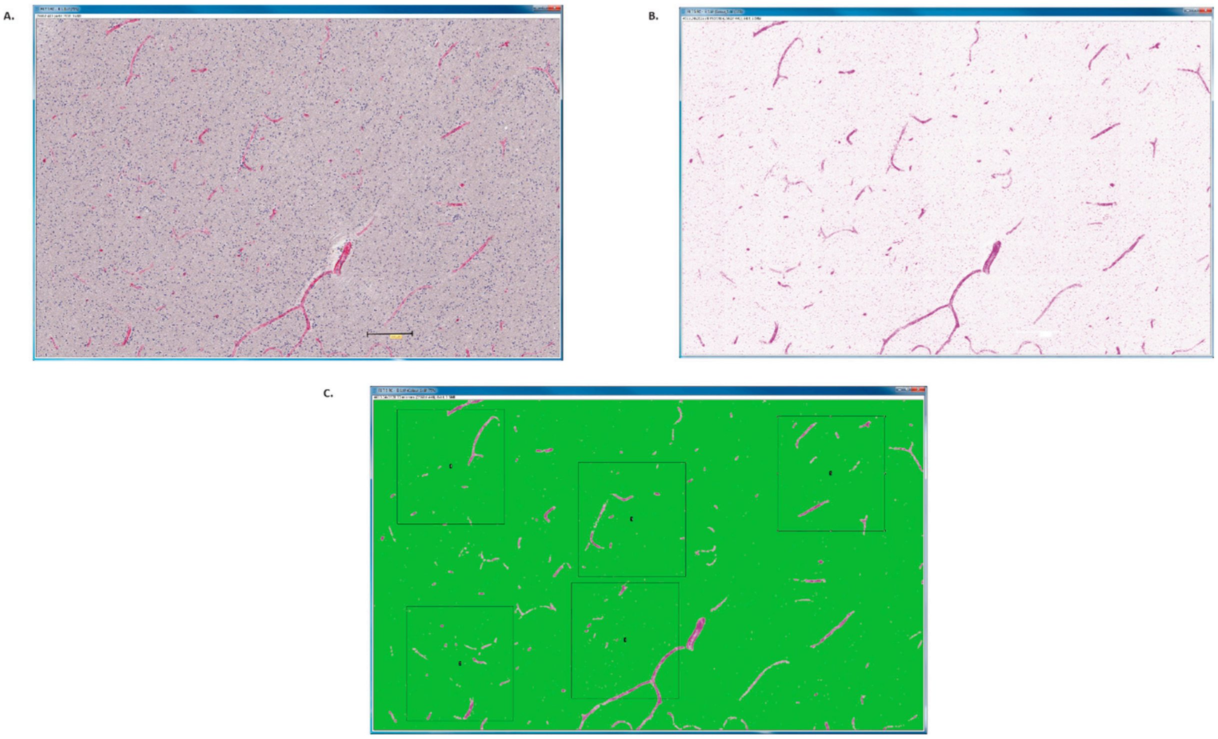

Figure S2. illustration of Quantification of percentage area of expression of the biomarkers.

Three different areas (fields of view at x200 magnification) with clear expression and microvascular density in the grey and white matter of each the brain regions used in the study were selected(A). The images were processed by color deconvolution using a set of macros instruction. The deconvoluted images (B) were smoothened and thresholded(C). 5 regions of interest (ROI) were chosen at random. In the ROIs(black boxes), the measurement of percentage area of the expression of these biomarkers was performed using “Measure percentage area” function on Image J.
